# Toward Epileptic Brain Region Detection Based on Magnetic Nanoparticle Patterning

**DOI:** 10.3390/s150924409

**Published:** 2015-09-22

**Authors:** Maysam Z. Pedram, Amir Shamloo, Aria Alasty, Ebrahim Ghafar-Zadeh

**Affiliations:** 1 Departement of Mechanical Engineering, Sharif University of Technology, Tehran, Iran; E-Mail: aalasti@sharif.edu; 2 Departement of Electrical Engineering and Computer Science, York University, Toronto, ON M3J1P3, Canada; E-Mail: maysam.pedram@gmail.com

**Keywords:** epilepsy, brain magnetic field, magnetic nanoparticle

## Abstract

Resection of the epilepsy foci is the best treatment for more than 15% of epileptic patients or 50% of patients who are refractory to all forms of medical treatment. Accurate mapping of the locations of epileptic neuronal networks can result in the complete resection of epileptic foci. Even though currently electroencephalography is the best technique for mapping the epileptic focus, it cannot define the boundary of epilepsy that accurately. Herein we put forward a new accurate brain mapping technique using superparamagnetic nanoparticles (SPMNs). The main hypothesis in this new approach is the creation of super-paramagnetic aggregates in the epileptic foci due to high electrical and magnetic activities. These aggregates may improve tissue contrast of magnetic resonance imaging (MRI) that results in improving the resection of epileptic foci. In this paper, we present the mathematical models before discussing the simulation results. Furthermore, we mimic the aggregation of SPMNs in a weak magnetic field using a low-cost microfabricated device. Based on these results, the SPMNs may play a crucial role in diagnostic epilepsy and the subsequent treatment of this disease.

## 1. Introduction

Epilepsy is a neurological disorder that leads to seizures. According to statistics, 30% of epileptic patients are refractory to all forms of medical treatment [1]. In the case of medically intractable focal epilepsy, the best treatment is resection of the foci [2]. In order to begin mapping of the epilepsy zone in the brain, magnetic resonance imaging [3], magnetoencephalography [4], single-photon emission computed tomography (SPECT) [5], positron emission tomography (PET) [6] or electroencephalography [7] are routinely used. As described in [4], incomplete mapping of the locations of epileptic neuronal networks results in incomplete resection of epileptic foci. Despite the fact that nanoparticles, particularly superparamagnetic nanoparticles (SPMNs), have attracted the attention of many researchers for the development of novel techniques for the detection of different diseases such as cancer and Alzheimer’s [8,9,10], less effort has been applied to nanoparticles for mapping the epilepsy zone. Among these, Akhtari *et al.* reported the advantages of functionalized nanoparticles for the detection of epilepsy using MRI [11]. MRI has the advantages of magnetic properties of the tissue at the sub-molecular level and can precisely construct images of the central nervous system (CNS) [12]. Therefore, MRI images may be enhanced by an injection of intravenous contrast agent. SPMNs can play the role of the contrast agent in an MRI to define and differentiate epileptic foci from the surrounding tissue. The detection of nanoparticles may also be performed using other techniques such as Superconducting Quantum Interference Device (SQUID) [13]. In the later paper, the weak magnetic field SQUID sensor is used for imaging the microscopic amount of nanoparticles by applying pulses to align the magnetic moment of nanoparticles. As described in this paper, this technique can efficiently be employed for breast cancer detection. However, the advantage of this technique for brain cancer detection in the presence of the magnetic activity of brain cells has not been studied.

**Figure 1:**
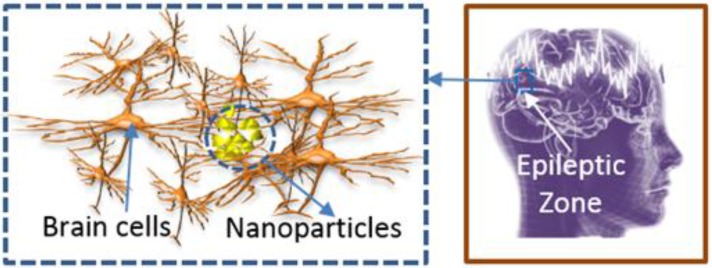
Illustration of SPMN’s aggregation in epileptic Zone.

In this paper, we demonstrate the benefits of the non-functionalized SPMNs as the contrast agent for the complete mapping of epilepsy location in the brain [12]. Indeed, the highly electrical activities of epileptic foci in the brain result in higher magnetic activities [14,15]. Therefore, the movement and aggregation of SPMNs can occur as illustrated in Figure 1. To date, many papers have described the electrical activities of neuronal networks in the brain [16,17,18] but less efforts have been made to study the magnetic behavior of neurons. Among these few works, A.S. Ferguson modeled a single neuron as a finite wire [19]. In this model, the magnetic field of each current point source represents transmembrane currents injected into the membrane. Based on these currents, the magnetic field around each wire was calculated. The focus of our paper is not the precise measurement of the magnetic field in the neurons. Indeed, by relying on high magnetic activity of epileptic cells, we aim to demonstrate the effect of such magnetic activities on nanoparticles for epilepsy detection purposes.

Despite the significant progress of drug delivery research, the delivery of large drug molecules through the blood-brain barrier (BBB) is still a major challenge. Due to the strong tight junctions, the endothelium patterning around the cerebral microvessels accurately controls the transportation of materials that are necessary for neural signaling. The permeability of BBB can be increased using various chemical techniques [20,21]. Furthermore, the physical techniques, such as high-frequency electromagnetic field radiation and ultrasonic techniques [22,23,24,25], have proven advantageous in increasing the permeability of the BBB. In this paper, however, we discuss the advantage of nanoparticles as an epilepsy detection method and not the delivery of nanoparticles through the BBB. Another challenge is the uniform distribution of nanoparticles. Along these lines, Sonavane *et al.* reported [26] that gold with nanoparticles of different sizes can be distributed uniformly in the brain. Masserini *et al.* [27] have put further efforts into this work by studying the advantage of gold material in penetrating uniformly into the brain. Therefore, the SPMNs with gold shell are the best candidate for transport in the brain. In this paper, we emphasize on the magnetic properties of SPMNs for epilepsy detection. For this reason, we have used bare SPMNs in the simulations and experiments.

## 2. Mathematical Models

As an electrical current passes through a metal wire, it causes a magnetic field. In a similar way, the intracellular and extracellular currents following through the neurons create magnetic fields in the brain. This complex brain magnetic field is the result of billions of nerve impulses. To date, many successful efforts have been made to obtain the information (strength, orientation, *etc.*) about the magnetic field in the brain by offering various theoretical models (e.g., monopoles [28]) and experimental platforms (e.g., magnetoencephalography (MEG) technique [4]). Based on these, neurons in the normal brain can produce a weak magnetic field (10fT-1pT) that is measurable with multichannel SQUID [29]. However, in the case of the epileptic brain, the extra electrical activities result in the higher magnetic field as demonstrated by the MEG technique [30,31]. In this paper, we aim to study the effect of the epileptic brain magnetic field on magnetic/superparamagnetic nanoparticles. In this study, we assumed that the nanoparticles are delivered into the brain and distributed uniformly. 

### 2.1. Creation of Magnetic Field on Neurons

Electrical activities are sustained and propagated via ionic currents through neuron membranes. Most of these transmembrane currents involve one of the four ionic species: sodium (Na^+^), potassium (K^+^), calcium (Ca^2+^), or chloride (Cl^−^). Based on the Hodgkin-Huxley model, and Poisson and Maxwell equations, we can estimate how these electrical activities result in creating a magnetic field (See Appendix A). In this study, we have engaged short wires mimicking the electrical currents to a number of neuron cells in a parallel direction. Let us assume such finite length wire sources that generate the following equation derived by using Biot-Savart law.

(1) $$B=\frac{\mu I}{2\pi \sqrt{{\left(y-a\right)}^{2}+{\left(z-b\right)}^{2}}}$$

where ***I*** is the neuronal current. ***y*** and ***z*** are the coordinates of the nanoparticle in 2D space. ***a***, ***b*** is also the location of the source point which is the distance between the nanoparticle and neurons. Indeed, by assuming that the lengths of these wires are much larger than the size of nanoparticles, Equation (1) can be derived from a general relationship of magnetic fields generated in a finite length of wires (see Appendix B). This simple model is used to produce the source point magnetic field as shown in the results section.

### 2.2. Effect of Magnetic Field and Brain Fluid on Superparamagnetic Nanoparticles (SPMNs)

The total applied force ($\vec{F}$) on an SPMN [32] consists of two components as shown in Equation (2).

(2) $$\vec{F}=\vec{F_{m}}+\vec{F_{D}}$$

The first component is the fluidic force $\vec{F_{D}}$, which is exerted by the suspending medium on a moving SPMN. The second component is the magnetic force $\vec{F_{m}}$, which is generated by the applied magnetic field gradient as shown in Equation (3) [33].

(3) $$\vec{F_{m}}=(\vec{m}.\nabla )\vec{B}=\frac{V\Delta \chi }{\mu_{0}}(\vec{B}.\nabla )\vec{B}$$

where $\Delta \chi =\chi -\chi_{CSF}$ is the effective susceptibility of the superparamagnetic nanoparticle relative to the Cerebrospinal fluid, μ0=4π×10−7NA2 and *V* is the magnetic permeability of free space and the volume of the nanoparticle, respectively.

The fluidic force for a spherical shape particle in a flow is determined by Stokes’s law [34],

(4) $$\vec{F_{D}}=-6\pi \eta r_{p}({\vec{v}}_{p}-\vec{u})$$

where $r_{p}$ is the radius of the particle, and η and $\vec{u}$ are the viscosity and velocity of the fluid, respectively. As physical parameters of CSF inside the brain are similar to those for water, the viscosity of the brain fluid is about η = 8.9 × 10^−4^ Ns/m^2^. It is noteworthy that other forces such as inertia, buoyancy, and gravitational forces as well as the interaction between particles can be neglected for low concentration of SPMNs in a fluid [35]. Therefore, using Newton’s law and merging Equations (2)–(4), we can obtain

(5) $$m_{p}\frac{d{\vec{v}}_{p}}{dt}=\vec{F_{m}}-6\pi \eta r_{p}({\vec{v}}_{p}-\vec{u})$$

where $m_{p}$ and ${\vec{v}}_{p}$ are the mass and velocity of the particle, respectively. This equation is efficiently used in our simulations in this paper to obtain the trajectories of moving nanoparticles shown in results’ section, described in Appendix C.

### 2.3. Aggregation of Nanoparticles

In this sub-section, we put forward a mathematical model to prove that the aggregation of nanoparticles occurs due to the magnetic field generated by neurons. For this purpose, we employ the potential energy function shown in Equation (6) [36].

(6) $$U=\frac{\mu_{0}}{4\pi }\{\frac{\vec{m_{a}}.\vec{m_{b}}}{{\left|\vec{r}\right|}^{3}}-\frac{3(\vec{m_{a}}.\vec{r})(\vec{m_{b}}.\vec{r})}{{\left|\vec{r}\right|}^{5}}\}$$

where $\vec{r}$ is the distance (or displacement) vector between two nanoparticles and $\vec{m_{a}}$ and $\vec{m_{b}}$ are the related magnetic dipole moments of nanoparticle *A* and *B* which can be obtained from the following equations.

(7) $$\begin{array}{l} \vec{m_{a}}=\chi \vec{B}(P_{A})\\ \vec{m_{b}}=\chi \vec{B}(P_{B}) \end{array}$$

where $P_{A}$ and $P_{B}$ are the positions of two particles at *A* and *B* points with the Cartesian coordinates of (x_A_, y_A_, z_A_) and (x_B_, y_B_, z_B_), respectively. $\vec{B}(P_{A})$ and $\vec{B}(P_{A})$ is magnetic flux density associated with $P_{A}$ and $P_{B}$. Also, χ is the coefficient associated with SPMNs. The magnetic flux density expressed in Equation (1) can also be used to obtain dipole moments as the function of coordinates.

(8) $$\begin{array}{lll} \vec{m_{a}}=\frac{\chi \mu_{0}I_{eff}}{2\pi }[\frac{-y_{A}a_{x}+x_{A}a_{y}}{r_{A}^{2}}], & & r_{A}^{2}=x_{A}^{2}+y_{A}^{2}\\ \vec{m_{b}}=\frac{\chi \mu_{0}I_{eff}}{2\pi }[\frac{-y_{B}a_{x}+x_{B}a_{y}}{r_{B}^{2}}], & & r_{B}^{2}=x_{B}^{2}+y_{B}^{2} \end{array}$$

where ***a_x_*** and ***a_y_*** are unit vectors in horizontal and vertical directions, respectively. Also, $I_{eff}$ is the effective equivalent current of the brain cells. By substituting $d=\sqrt{{\left(x_{A}-x_{B}\right)}^{2}+{\left(y_{A}-y_{B}\right)}^{2}}$, $\vec{m_{a}}$ and $\vec{m_{b}}$ we can derive the function as expressed in Equation (9) (see Appendix B).

(9) $$U≃\frac{\gamma^{2}\chi^{2}}{r_{A}^{2}r_{B}^{2}}\left[-\frac{3{\left(x_{B}y_{A}+x_{A}y_{B}\right)}^{2}}{d^{5}}\right]$$

**Figure 2:**
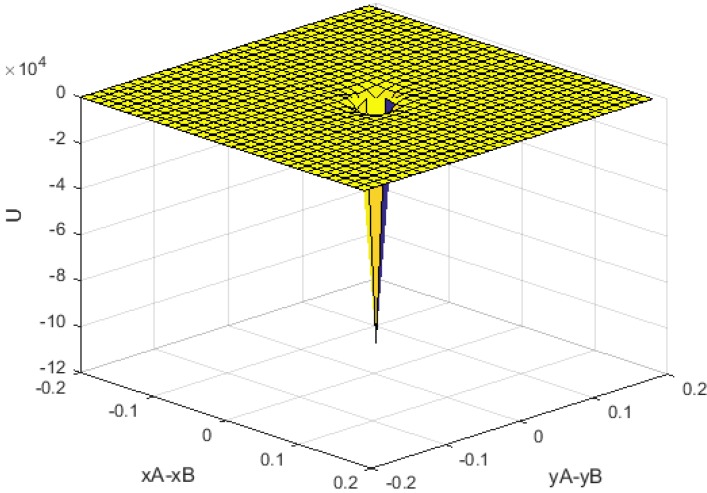
Numerically analyzed potential energy (units in axis x-y are *m*, unit in z-axis is *J*).

Figure 2 illustrates the U function where numerical values of *γ*, *χ* are a positive parameter function of current and permeability. As seen in this figure, the minimum value of *U* occurs when (x_A_, y_A_) → (x_B_, y_B_).

## 3. Two-Dimensional (2D) Simulation Results

**Figure 3:**
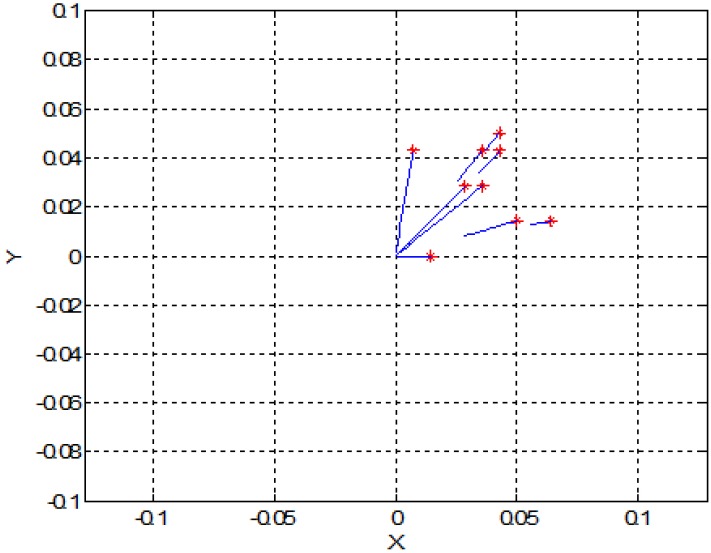
Trajectory of the nine nanoparticles in the effect of one magnetic field source (units are mm).

**Figure 4:**
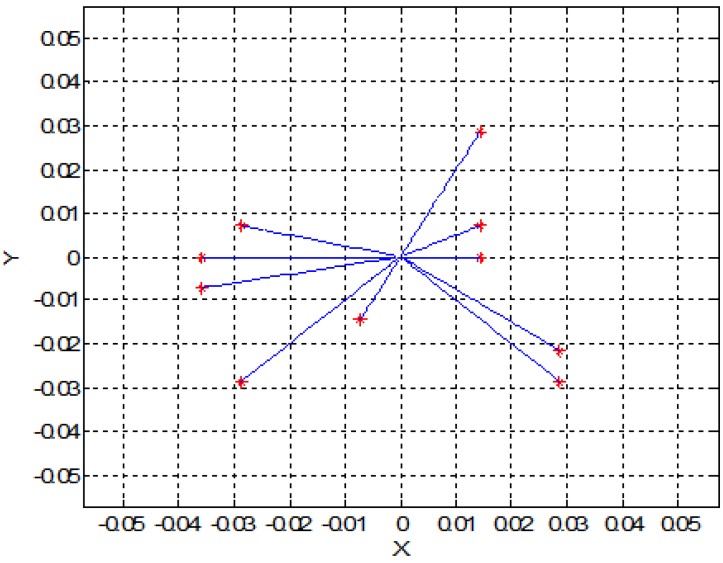
Trajectory of ten nanoparticles in the effect of one magnetic field source (units are mm).

In this section, for simplicity, we present a 2D simulation of SPMNs under the effect of the magnetic field. Based on Equation (5) and energy of SPMNs, the trajectory of each nanoparticle has been derived. In the previous section, we put forward a mathematical proof of aggregation. In this method, based on the polar system, the trajectory of each nanoparticle can be obtained. These simulations have been done in MATLAB. As it shows in Figure 3 and Figure 4, nine nanoparticles move toward the center of the magnetic field source. 

**Figure 5:**
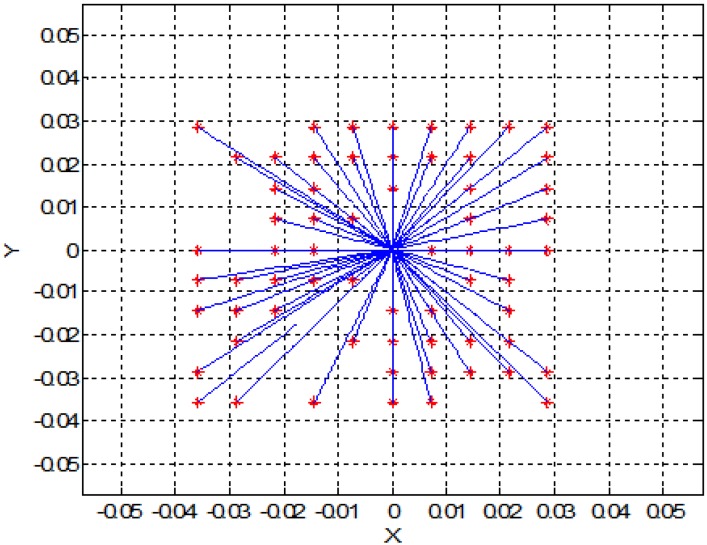
Trajectory of seventy nanoparticles under one magnetic field source (units are mm).

**Figure 6:**
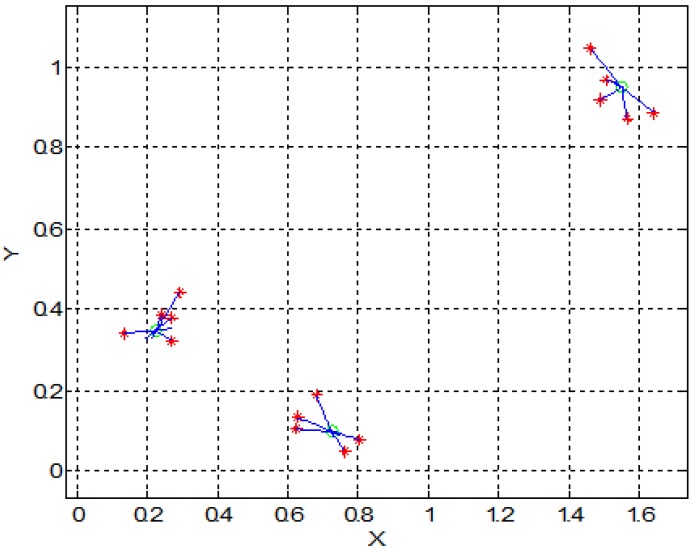
Trajectory of fifteen nanoparticles under three magnetic field sources (units are mm).

**Figure 7:**
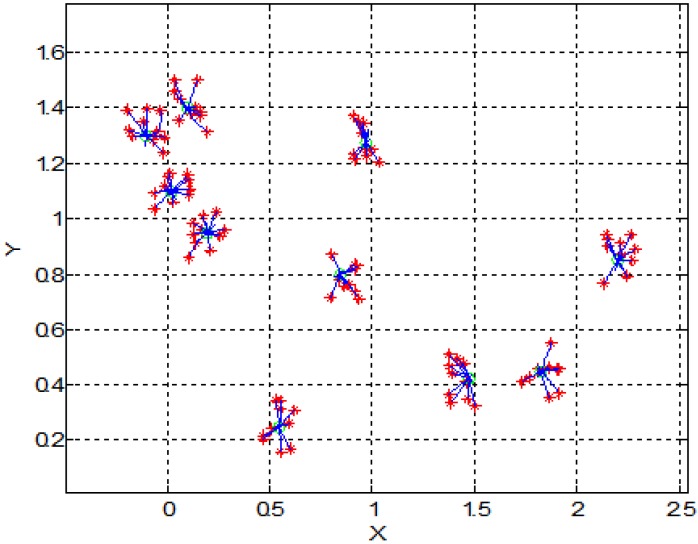
Trajectory of 100 nanoparticles under ten different magnetic field sources (Units of X and Y axes are mm).

Figure 5 shows the effect of magnetic field on a larger number of SPMNs. It is noteworthy that the movement of each nanoparticle is independent of other nanoparticles. However, all nanoparticles move toward the same center. The magnetic effect of the neuronal network can be simulated with a number of magnetic sources. For instance, in Figure 6 and Figure 7, the behavior of SPMNs under the effect of three and ten different epileptic sources are illustrated.

**Figure 8:**
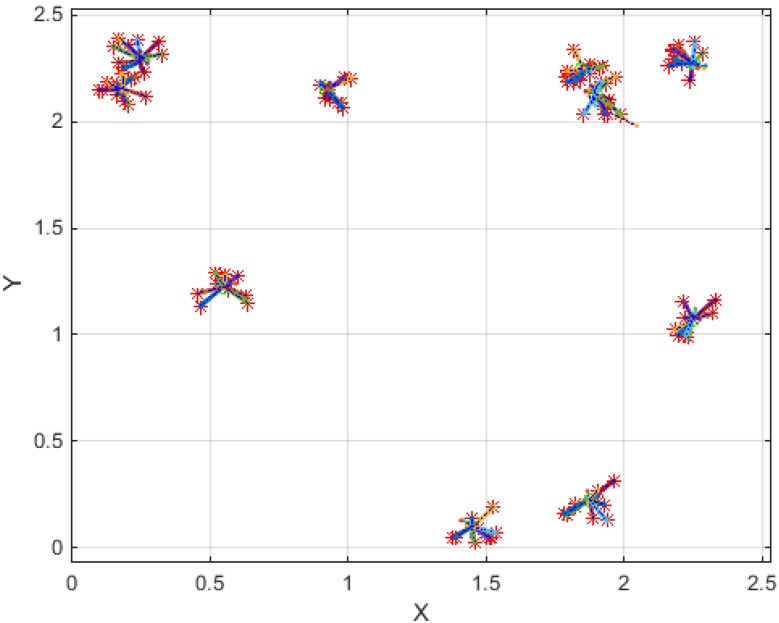
Trajectory of 100 nanoparticles under ten different magnetic field sources (Units of X and Y axes are mm).

As seen in Figure 6, Figure 7 and Figure 8, the SPMNs are aggregated over the closest source of the magnetic field. It is noteworthy that the magnetic fields in small neuronal circuitries in the brain are a function of time and space. However, the effect of these magnetic fields over time can be modeled with a DC magnetic field. The simulation results show that the local SPMNs near focal epilepsy can be aggregated. The magnetic field in epileptic foci is calculated by changing the amplitude and frequency of *I_eff_* in Equation (8). 

## 4. Experimental Model and Results

In this section, we discuss the experimental results demonstrating a weak magnetic field generated in the microfabricated coil.

### 4.1. Experimental Setup

**Figure 9:**
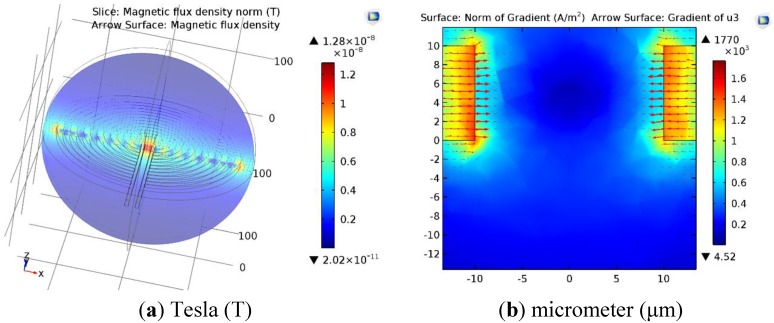
COMSOL simulation of the magnetic field above the micro coil; Gradient of the magnetic field (**a**) from the top and (**b**) close to the conductor.

The experimental setup includes a micro coil realized on glass using Indium tin oxide (ITO) (Delta Technologies, Limited, Loveland, CO, USA), a current source (Keithy 2400), and microscope to observe the clusters of SPMNs (3327NG Iron Oxide Nanoparticles, SkySpring Nanomaterials, Inc., Houston, TX, USA), 10~15 nm, spherical, 43.8 emu/g saturation magnetization). To pattern ITO, a photolithography technique is used to create a microcoil. As seen in Figure 9. In order to increase the strength of a magnetic field on the surface of the glass, all turns of the micro coil are kept in parallel. The nanoparticles are distributed on the surface of the glass, and the magnetic field is exposed to nanoparticles.

### 4.2. COMSOL Simulation Results of Micro Coils

The COMSOL simulation results in Figure 9a,b demonstrate that the maximum gradient of the magnetic field occurs in the center of the micro coil. The parallel design of the micro coil is the reason behind the creation of a magnetic force toward the center. In fact, by creating this structure, the strength and gradient of the magnetic field are maximized in the middle. As seen in Figure 9b, the gradient of the magnetic field is at a maximum near the micro coil. In these simulations, the thickness and size of the electrode is similar to the microfabricated micro coil.

### 4.3. Experimental Results

**Figure 10:**
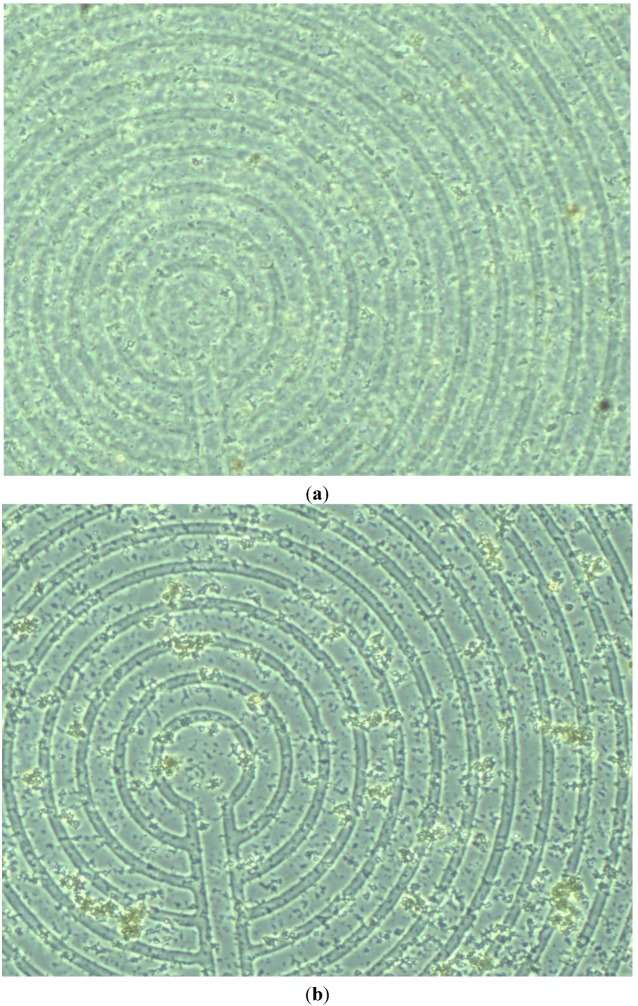

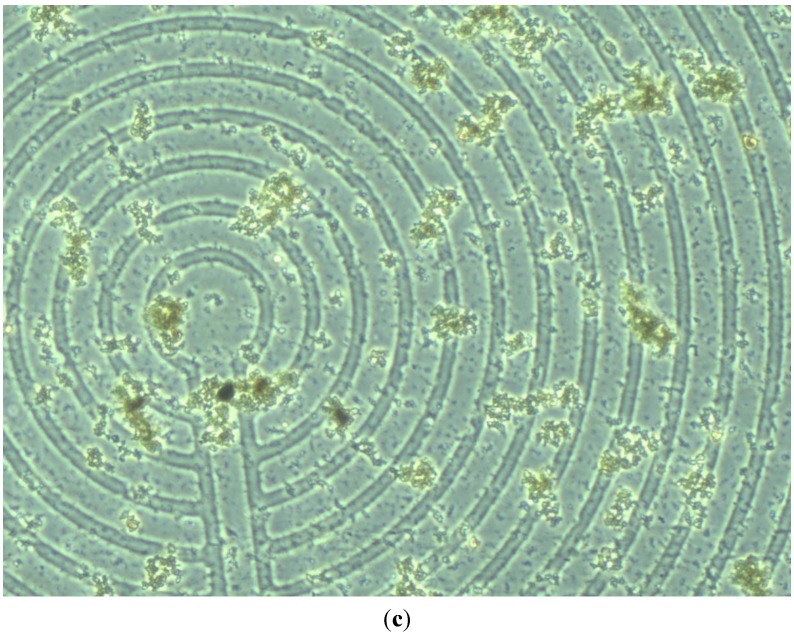
Experimental results: aggregation of nanoparticles above the microcoil. Generating a magnetic field (**a**) before applying an electromagnetic field; (**b**) immediately after applying electromagnetic field and (**c**) 10 s after applying electromagnetic field.

By applying a weak current (~1 μA), a weak magnetic field (~2 pT) is generated. Therefore, as expected from Equation (1), the aggregation of SPMNs occurs. Based on the COMSOL simulation results, the magnetic field above the surface of the glass is in the order of (2 pT to 10 nT). The aggregation is achieved by this magnetic field after about less than a minute for all nanoparticles. Figure 10a shows the microscopic image of micro coils underneath the nanoparticles at different times. As it is shown in Figure 10b, the aggregation happens immediately after applying a magnetic field. The clusters of nanoparticles become very large and they can be seen under the optical microcope after about 10 s as seen in Figure 10c. The disaggregation occurs after disconnecting the current source from micro coils.

Based on the experimental results presented in Figure 10, the maximum gradient can be seen in the center of the micro coil. However, the aggregates can be seen all around the micro coil. This is in agreement with the simulation results shown in Figure 3, Figure 4, Figure 5, Figure 6, Figure 7 and Figure 8. Indeed, the nanoparticles move in the short distances to create aggregates. Once the small aggregates are generated, they require higher magnetic forces to move. Therefore, the gradient of the magnetic field is not enough to displace all aggregates toward the center where the magnetic field is at its maximum.

## 5. Discussion

In this section, we briefly discuss the practical considerations associated with the proposed method of epilepsy detection.

*Aggregation Parameters:* The time scale for reorientation and aggregation of nanoparticles depends on various parameters including the strength of magnetic field, the distance of the nanoparticle from the epileptic source and the size of nanoparticles. Based on the simulation results, for an epileptic source with a 2 pT magnetic field, the aggregation of 10~15 nm-sized nanoparticles occurs in a circular area with a diameter equal to 300 micron in 5~10 s. Furthermore, the viscosity of brain tissue in the proximity of the epileptic source is another important factor affecting the time of aggregation and the minimum magnetic field to generate clusters with a specific size.

*Distribution of Nanoparticles in Brain:* The uniform distribution of SPMNs enables the detection of epilepsy foci generated anywhere in the brain. The uniform distribution of nanoparticles into the brain is a key challenge that has been addressed by researchers. Among these researchers, Sonavane *et al.* reported that the gold nanoparticles with different sizes could be distributed uniformly in the brain [26]. Masserini *et al.* [27] have built on this study by investigating the advantages of gold material to penetrate uniformly into the brain. Based on these results, the SPMNs with gold shell are the best candidate for uniform distribution and improved permeability purposes. 

*MRI-Guided Epilepsy Detection:* As already mentioned, MRI is the best method to detect epilepsy in the deep brain using SPMNs. However, an MRI can generate a high strength and high gradient magnetic field that may result in the aggregation of SPMNs. For this reason, in our protocol, the lack of MRI field and gradient effects on clusters suggest that MR images should be acquired postictally (during the interictal stage). In this case, let us assume the aggregation occurs during the ictal state with high epileptic activity. The clusters with a large number of iron oxide atoms are generated during this phase. Therefore, despite the effect of the MRI on the aggregation of single SPMNs, the magnetic field generated by the MRI is not sufficient to cause movement of the heavy clusters.

In theory, the MRI field and gradients have the same ability as any other external magnetic field to cause the aggregation of SPMNs. However, in practice, by optimizing the size and shape of the SPMNs, it is possible to prevent the aggregation of SPMNs exposed to the MRI field and gradient. Additionally, it is assumed that the aggregation and imaging are performed in two separate phases. The first phase includes the aggregation of SPMNs due to high electrical and magnetic activities of epileptic cells. In the second phase, the MRI will be performed to demonstrate the boundary of the epileptic region. Therefore, the MRI field and gradient cannot effectively result in the movement of large SPMN aggregates generated in the first phase.

*Disaggregation of SPMNs:* It is expected that SPMNs disaggregate immediately after an epilepsy attack. However, the hysteresis of iron oxide nanoparticles does not allow the disaggregation to occur rapidly. Similar to aggregation, the disaggregation also depends on several other parameters, specifically, the charges of nanoparticles. Indeed, the disaggregation allows enough time for the MRI to be used for the observation of clusters. Based on the preliminary experiments described in the previous sections, the disaggregation process starts immediately after disconnecting the electrical current from micro coils. In this process the poorly connected nanoparticles are removed from the cluster immediately. However, many nanoparticles remain connected to the cluster, and a sufficient amount of time is required after the current disconnection for the disaggregation of entire SPMNs.

*The Shape of Nanoparticles:* Despite the fact that the gradient of the magnetic field is an important factor in generating force on sphere-shape nanoparticles, the rod-shaped nanoparticles can be used in the very low gradient field ranging from the DC magnetic field. In this study, we have focused on sphere-shaped nanoparticles. Based on our simulation results, the limiting gradient is about 2 pT/um. However, this value of the gradient of the magnetic field depends on the size of nanoparticles and other factors as well.

*Current and Future Works:* As described in the previous sections, this paper outlined the advantage of SPMNs for epilepsy detection. Based on the preliminary experimental results using microfabricated micro coils, the SPMNs can aggregate in a low magnetic field. This paper also suggested that the SPMNs delivered into the brain could be used as an MRI contrast agent for the detection of epilepsy foci. However, several questions remain unanswered and further steps should be taken in this research approach toward a safe and practical clinical protocol. For instance, further simulations and experiments should be performed in order to determine the minimum size of detectable epileptic foci, the minimum required time for the aggregation of SPMNs, the maximum disaggregation time for MRI purposes and other related critical parameters. Although the aforementioned preliminary results in this paper can shed the light on novel technology development for epilepsy foci detection, the optimization of SPMNs as the new MRI contrast agent for epilepsy detection should become the focus of future research. 

## 6. Conclusions

In this paper, we introduced a novel technique for the detection of epileptic foci. In this new approach, the SPMNs play a significant role as a novel MRI contrast agent for the epilepsy detection. This new technique relies on the aggregation of SPMNs in the brain due to the high magnetic activity of the neural network in the epilepsy zone. Herein we developed mathematical and simulation platforms to prove the concept. We also have demonstrated and discussed experimental results by mimicking the effect of weak epileptic foci *in vitro* using a low-cost experimental setup. Based on simulation and experimental results, SPMNs can aggregate in the brain and consequently enhance the related MRI images from the epileptic region. As the continuation of this work, we will develop *in vivo* experiments using animal models.

## Figures and Tables

**Figure B1:**
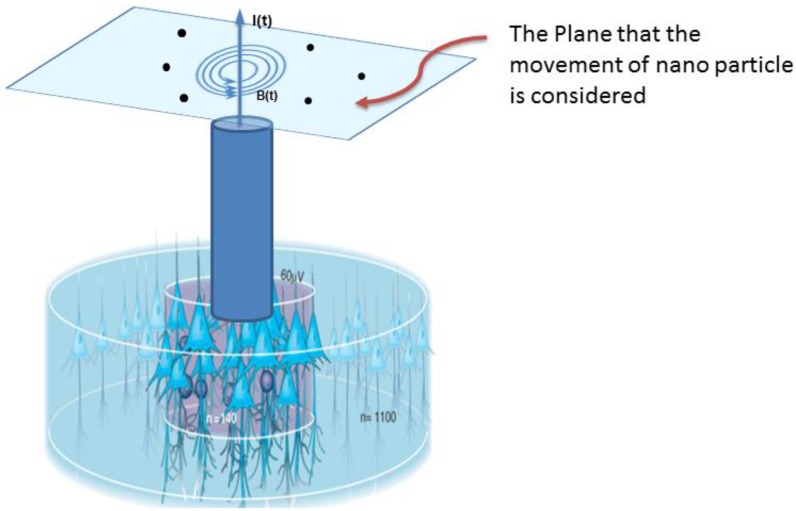
Schematic of 2D analysis of motion of nanoparticles (Nanoparticle movement is considered in *y* and *z* plane).

**Figure B2:**
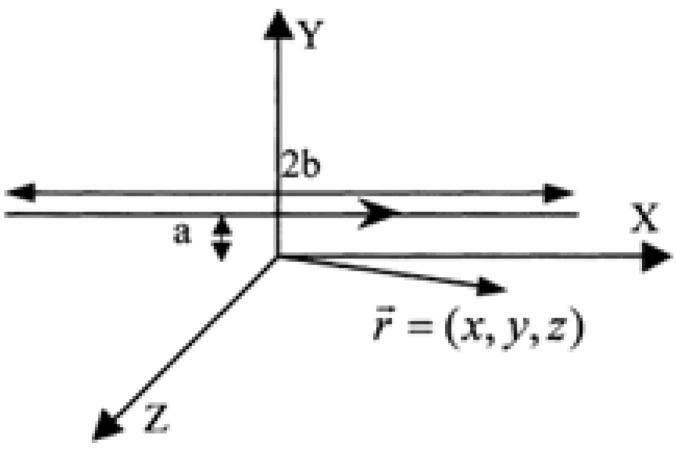
Coordination of single wire in three-dimensional space.

## Appendix

### A. Electromagnetic Activities of Brain

Electrical activity in neurons is sustained and propagated via ionic currents through neuron membranes. Most of these transmembrane currents involve one of the four ionic species: sodium (Na^+^), potassium (K^+^), calcium (Ca^2+^), or chloride (Cl^−^). Based on the Hodgkin-Huxley model [37], there is a relationship between the electrical potential.

(A1) $$C\dot{V}=I-{\bar{g}}_{K}n^{4}\left(E-E_{K}\right)-{\bar{g}}_{Na}m^{3}h\left(E-E_{Na}\right)-g_{L}\left(V-E_{L}\right)$$

where *V* is membrane potential, *I* is total membrane current per unit area, *C* is membrane capacitor per unit area and n, m and h can be obtained from the following equations.

(A2) $$\dot{n}=\alpha_{n}\left(V\right)\left(1-n\right)-\beta_{n}\left(V\right)n$$

(A3) $$\dot{m}=\alpha_{m}\left(V\right)\left(1-m\right)-\beta_{m}\left(V\right)m$$

(A4) $$\dot{h}=\alpha_{h}\left(V\right)\left(1-h\right)-\beta_{h}\left(V\right)h$$

where $\alpha_{n},\alpha_{m},\alpha_{h}$ can be obtained from the following equations.

(A5) $$\alpha_{n}\left(V\right)=0.01\frac{10–V}{\text{exp}\left(\frac{10–V}{10}\right)–1},\;\beta_{n}\left(V\right)=0.125\text{exp}\left(\frac{–V}{80}\right)$$

(A6) $$\alpha_{m}\left(V\right)=0.1\frac{25–V}{\text{exp}\left(\frac{25–V}{10}\right)–1},\;\beta_{m}\left(V\right)=4\text{exp}\left(\frac{–V}{80}\right)$$

(A7) $$\alpha_{h}\left(V\right)=0.07exp\left(\frac{–V}{20}\right),\;\beta_{h}\left(V\right)=\frac{1}{\text{exp}\left(\frac{30–V}{10}\right)+1}$$

Also reverse potentials and maximal conductances are achieved from the following equations.

$$E_{k}=-12\;\text{mV},E_{L}=10.6\;\text{mV},E_{Na}=120\;\text{mV}$$

$${\bar{g}}_{K}=36\;\text{mS}/{\text{cm}}^{2},{\bar{g}}_{Na}=120\;\text{mS}/{\text{cm}}^{2},\;{\bar{g}}_{L}=0.3\;\text{mS}/{\text{cm}}^{2}$$

where $E_{k},E_{Na},E_{L}$ are the reverse potential of K, Na and Cl ionic channels and ${\bar{g}}_{K},{\bar{g}}_{Na},{\bar{g}}_{L}$ are the maximal value of the conductance of K, Na and Cl ionic channels, respectively. Equations (A1)–(A7) and all time variant parameters express the electrical potential on the surface of neuronal membranes. By using these values as a boundary condition of Equation (A1), spatial distribution of electrical potential is achieved.

(A8) $$\nabla^{2}V=\frac{\rho_{V}}{\varepsilon }$$

where, $\rho_{V}$ is free charge density, and ε is permittivity of the medium. $\rho_{V}$ is related to the distribution of K, Na and Cl in the medium. Electrical potential and electrical field are mapped together based on Equation (A9).

(A9) E=−∇V

In this equation, *V* represents the spatial distribution of electrical potential. By applying gradient vector on *V*, an electrical field is derived. Since the electrical activity of neurons is time dependent, based on Maxwell equations, a magnetic field is orthogonally spread around electrical fields through space. Equations (A10) and (A11) are shown this behavior [38].

(A10) $$\nabla \times E=-\frac{\partial B}{\partial t}$$

(A11) $$\nabla \times H=\frac{\partial D}{\partial t}+J$$

where, $D,\rho_{V},B,H$ and *J* are the electrical flux density, electric charge density, magnetic flux density, electrical field, magnetic field, and electric current density, respectively. The Maxwell equation can be solved analytically and numerically [39].

### B. Electromagnetic Field of Finite Length Wires

The electrical and magnetic activity of neuron cells are the functions of time and space. Since, in this paper, we aim to show the effect of seizure on the SPMNs delivered in the brain, the 2D model can be employed to study the magnetic effect of a number of neuron cells in a parallel direction as shown in Figure B1. The total generated by the magnetic field is related to all current density flowing through a virtual circle around neurons.

The formulation of the magnetic field generated by finite length wire (Figure B2), is derived from Equation (5).

(B1) $$B_{x}=0$$

(B2) $$B_{y}=-\frac{\mu zI}{4\pi [{\left(a-y\right)}^{2}+z^{2}]}\times \left[\frac{b-x}{\sqrt{{\left(b-x\right)}^{2}+{\left(a-y\right)}^{2}+z^{2}}}+\frac{b+x}{\sqrt{{\left(b+x\right)}^{2}+{\left(a-y\right)}^{2}+z^{2}}}\right]$$

(B3) $$B_{z}=-\frac{\mu (a-y)I}{4\pi [{\left(a-y\right)}^{2}+z^{2}]}\times \left[\frac{b-x}{\sqrt{{\left(b-x\right)}^{2}+{\left(a-y\right)}^{2}+z^{2}}}+\frac{b+x}{\sqrt{{\left(b+x\right)}^{2}+{\left(a-y\right)}^{2}+z^{2}}}\right]$$

where *µ*, 2*b*, *a* are permeability, the length of wire and drift from the origin on the axis, respectively. *I* is the current flowing through the wire and *x*, *y*, *z* are arbitrary positions in space. As the length of these wires is much larger than the size of nanoparticles, the magnetic fields generated by these wires can be calculated by the following relationships.

(B4) $$B_{x}=0$$

(B5) $$B_{y}=-\frac{zI}{2\pi [{\left(a-y\right)}^{2}+z^{2}]}$$

(B6) $$B_{z}=-\frac{(a-y)I}{2\pi [{\left(a-y\right)}^{2}+z^{2}]}$$

(B7) $$\sqrt{{B^{2}}_{y}+{B^{2}}_{z}}=B_{\theta }=\frac{\mu I}{2\pi r}$$

where *r* is the distance between the wire and the point (x = 0, y, z).

### C. U Function

Let us calculate $\vec{r},d$ and the magnetic fields in Equation (6) as expressed in Equations (C1)–(C8):

(C1) $$\vec{r}=(x_{B}-x_{A})\vec{a_{x}}+(y_{B}-y_{A})\vec{a_{y}}$$

(C2) $$d=\sqrt{{\left(x_{B}-x_{A}\right)}^{2}+{\left(y_{B}-y_{A}\right)}^{2}}$$

(C3) $$\vec{B_{A}}=\frac{\mu_{0}I_{eff}}{2\pi }[\frac{-y_{A}\vec{a_{x}}+x_{A}\vec{a_{y}}}{r_{A}^{2}}]$$

(C4) $$r_{A}^{2}=x_{A}^{2}+y_{A}^{2}$$

(C5) $$\vec{B_{B}}=\frac{\mu_{0}I_{eff}}{2\pi }[\frac{-y_{B}\vec{a_{x}}+x_{B}\vec{a_{y}}}{r_{B}^{2}}]$$

(C6) $$r_{B}^{2}=x_{B}^{2}+y_{B}^{2}$$

where $\vec{B_{A}}$ and $\vec{B_{B}}$ are magnetic flux density in points *A* and *B* and $\vec{a_{x}}$ and $\vec{a_{y}}$ are unit vectors in the horizontal and vertical directions, respectively. Also, $I_{eff}$ is the effective equivalent current of the brain cells. By substituting all equations into the potential energy equation, Equation (C7) is concluded.

(C7) $$U=\frac{\gamma^{2}\chi^{2}}{r_{A}^{2}r_{B}^{2}}\left[\frac{\left(x_{A}x_{B}+y_{A}y_{B})({\left(x_{B}-x_{A}\right)}^{2}+{\left(y_{B}-y_{A}\right)}^{2}\right)}{d^{5}}-\frac{3{\left(x_{B}y_{A}+x_{A}y_{B}\right)}^{2}}{d^{5}}\right]$$

where *γ*, *χ* are a positive parameter function of current and permeability. By simplifying Equations (C7) and (C8), the following isobtained:

(C8) $$U≃\frac{\gamma^{2}\chi^{2}}{r_{A}^{2}r_{B}^{2}}\left[-\frac{3{\left(x_{B}y_{A}+x_{A}y_{B}\right)}^{2}}{d^{5}}\right]$$

## References

[1] Ben-Menachem E., Henriksen O., Dam M., Mikkelsen M., Schmidt D., Reid S., Reife R., Kramer L., Pledger G., Karim R. (1996). Double-Blind, Placebo-Controlled Trial of Topiramate as Add-on Therapy in Patients with Refractory Partial Seizures. Epilepsia.

[2] Rosenow F., Lüders H. (2001). Presurgical evaluation of epilepsy. Brain.

[3] Woermann F., Free S.L., Koepp M.J., Sisodiya S.M., Duncan J.S. (1999). Abnormal cerebral structure in juvenile myoclonic epilepsy demonstrated with voxel-based analysis of MRI. Brain.

[4] Iwasaki M., Pestana E., Burgess R.C., Lüders H.O., Shamoto H., Nakasato N. (2005). Detection of epileptiform activity by human interpreters: Blinded comparison between electroencephalography and magnetoencephalography. Epilepsia.

[5] Halama J., Henkin R. (1986). Single photon emission computed tomography (SPECT). Freeman and Johnson’s Clinical Radionuclide Imaging.

[6] Ter-Pogossian M.M. (1983). Positron emission tomography (PET). Diagnostic Imaging in Medicine.

[7] Claassen J., Mayer S.A., Kowalski R.G., Emerson R.G., Hirsch L.J. (2004). Detection of electrographic seizures with continuous EEG monitoring in critically ill patients. Neurology.

[8] Georganopoulou D.G., Chang L., Nam J.M., Thaxton C.S., Mufson E.J., Klein W.L., Mirkin C.A. (2005). Nanoparticle-based detection in cerebral spinal fluid of a soluble pathogenic biomarker for Alzheimer’s disease. Proc. Natl. Acad. Sci. USA.

[9] Jordan A., Scholz R., Wust P., Fähling H., Felix R. (1999). Magnetic fluid hyperthermia (MFH): Cancer treatment with AC magnetic field induced excitation of biocompatible superparamagnetic nanoparticles. J. Magn. Magn. Mater..

[10] Brigger I., Dubernet C., Couvreur P. (2002). Nanoparticles in cancer therapy and diagnosis. Adv. Drug Deliv. Rev..

[11] Akhtari M., Bragin A., Cohen M., Moats R., Brenker F., Lynch M.D., Vinters H.V., Engel J. (2008). Functionalized magnetonanoparticles for MRI diagnosis and localization in epilepsy. Epilepsia.

[12] Brown R.W., Haacke E.M., Thompson M.R., Venkatesan R. (2014). Magnetic Resonance Imaging: Physical Principles and Sequence Design.

[13] Flynn E., Bryant H. (2005). A biomagnetic system for *in vivo* cancer imaging. Phys. Med. Biol..

[14] Wallace E., Benayoun M., van Drongelen W., Cowan J.D. (2011). Emergent oscillations in networks of stochastic spiking neurons. PLoS ONE.

[15] Lee H., Hereld M., Stevens R., van Drongelen W. (2005). Epileptiform Activity Patterns in Coupled Neuronal Networks. Int. J. Bioelectromag..

[16] Hodgkin A.L., Huxley A.F. (1952). A quantitative description of membrane current and its application to conduction and excitation in nerve. J. Physiol..

[17] Brzychczy S., Poznanski R.R. (2013). Mathematical Neuroscience.

[18] Terman D. (2014). Mathematical Neuroscience. Am. Math. Mon..

[19] Ferguson A.S. (1991). Theoretical Calculation of Magnetic Fields Generated by Neural Currents.

[20] Jolesz F.A. (2014). Science to Practice: Opening the Blood-Brain Barrier with Focused Ultrasound—A Potential Treatment for Alzheimer Disease?. Radiology.

[21] Kinoshita M., McDannold N., Jolesz F.A., Hynynen K. (2006). Noninvasive localized delivery of Herceptin to the mouse brain by MRI-guided focused ultrasound-induced blood–brain barrier disruption. Proc. Natl. Acad. Sci. USA.

[22] Kinoshita M., McDannold N., Jolesz F.A., Hynynen K. (2006). Targeted delivery of antibodies through the blood–brain barrier by MRI-guided focused ultrasound. Biochem. Biophys. Res. Commun..

[23] Lampropoulos N., Karvelas E., Sarris I. (2015). Computational Modeling of an MRI Guided Drug Delivery System Based on Magnetic Nanoparticle Aggregations for the Navigation of Paramagnetic Nanocapsules.

[24] Latulippe M., Martel S. Dipole Field Navigation for targeted drug delivery. Proceedings of the 2014 5th IEEE RAS & EMBS International Conference on Biomedical Robotics and Biomechatronics.

[25] Liu X., Tu M., Kelly R.S., Chen C., Smith B.J. (2004). Development of a computational approach to predict blood-brain barrier permeability. Drug Metab. Dispos..

[26] Sonavane G., Tomoda K., Makino K. (2008). Biodistribution of colloidal gold nanoparticles after intravenous administration: Effect of particle size. Colloids Surf. B.

[27] Masserini M. (2013). Nanoparticles for brain drug delivery. ISRN Biochem..

[28] Nunez P.L., Srinivasan R. (2006). Electric Fields of the Brain: The Neurophysics of EEG.

[29] Hämäläinen M., Hari R., Ilmoniemi R.J., Knuutila J., Lounasmaa O.V. (1993). Magnetoencephalography—Theory, instrumentation, and applications to noninvasive studies of the working human brain. Rev. Modern Phys..

[30] Barkley G.L., Baumgartner C. (2003). MEG and EEG in epilepsy. J. Clin. Neurophysiol..

[31] Iida K., Hashizume A., Otsubo H. (2015). MEG and Magnetic Source Imaging in MRI-Negative Refractory Focal Epilepsy. MRI-Negative Epilepsy: Evaluation and Surgical Management.

[32] Cao Q., Han X., Li L. (2012). Numerical analysis of magnetic nanoparticle transport in microfluidic systems under the influence of permanent magnets. J. Phys. D.

[33] Guo S., Deng Y.L., Zhao L.B., Chan H.L.W., Zhao X.Z. (2008). Effect of patterned micro-magnets on superparamagnetic beads in microchannels. J. Phys. D.

[34] Pankhurst Q.A., Connolly J., Jones S.K., Dobson J.J. (2003). Applications of magnetic nanoparticles in biomedicine. J. Phys. D.

[35] Haverkort J., Kenjereš S., Kleijn C. (2009). Magnetic particle motion in a Poiseuille flow. Phys. Rev. E.

[36] Griffiths D.J., College R. (1999). Introduction to Electrodynamics.

[37] Izhikevich E.M., Moehlis J. (2008). Dynamical Systems in Neuroscience: The geometry of excitability and bursting. SIAM Rev..

[38] Corson D.R., Lorrain P. (1962). Introduction to Electromagnetic Fields and Waves.

[39] Yee K.S. (1966). Numerical solution of initial boundary value problems involving Maxwell’s equations in isotropic media. IEEE Trans. Antennas Propag..

